# Endoscopic Submucosal Dissection for Gastric Tube Carcinoma after Esophagectomy Contributes to Long-Term Outcomes

**DOI:** 10.1155/2022/1631415

**Published:** 2022-02-10

**Authors:** Satoru Hashimoto, Hiroki Sato, Ken-ichi Mizuno, Kazuya Takahashi, Masafumi Takatsuna, Junji Yokoyama, Hiroshi Ichikawa, Manabu Takeuchi, Masaaki Kobayashi, Shuji Terai

**Affiliations:** ^1^Division of Gastroenterology and Hepatology, Graduate School of Medical and Dental Sciences, Niigata University, 1-757 Asahimachi-dori, Chuo-ku, Niigata-City, Niigata 951-8510, Japan; ^2^Department of Gastroenterology, Saiseikai Kawaguchi General Hospital, 5-11-5 Nishikawaguchi, Kawaguchi-City, Saitama 332-8558, Japan; ^3^Department of Endoscopy, Niigata University Medical and Dental Hospital, 1-757 Asahimachi-dori, Chuo-ku, Niigata-city, Niigata 951-8510, Japan; ^4^Division of Digestive and General Surgery, Graduate School of Medical and Dental Sciences, Niigata University, 1-757 Asahimachi-dori, Chuo-ku, Niigata-City, Niigata 951-8510, Japan; ^5^Department of Gastroenterology, Nagaoka Red Cross Hospital, 2-297-1 Sensyu, Nagaoka-City, Niigata 940-2085, Japan; ^6^Department of Gastroenterology, Niigata Cancer Center Hospital, 2-15-3 Kawagishicho, Chuo-ku, Niigata-City, Niigata 951-8566, Japan

## Abstract

The incidence of gastric tube carcinoma (GTC) after esophagectomy for esophageal carcinoma has increased in recent years. Surgical removal of the reconstructed gastric tube is associated with high mortality, and endoscopic submucosal dissection (ESD) is a promising alternative. There are limited reports of ESD for GTC. This study investigated the efficacy and safety of ESD in GTC. This single-center retrospective study examined patients who underwent ESD for GTC after esophagectomy at our institution between 2003 and 2018. The curability of GTC with ESD was evaluated histologically according to the Japanese Gastric Cancer Treatment Guidelines. Patient characteristics and procedural and long-term outcomes were analyzed. Overall, 31 patients (29 men and 2 women; median age, 73 years) with 45 GTC lesions underwent ESD. The mean period between primary esophagectomy and the diagnosis of GTC was 10.6 years. Bleeding during ESD was noted in two patients (6.5%). No other adverse or fatal events such as perforation were noted. Complete resection and curative resection were documented in 80.6% and 48.4% of cases, respectively. The 3-year and 5-year overall survival rates were 67.6% and 47.7%, respectively. The 3-year and 5-year disease-specific survival rates were 100% and 92.9%, respectively. One patient died of GTC, and fourteen patients died of other diseases, including primary carcinoma in five cases. ESD was safe and provided good long-term outcomes in patients with GTC. Regular long-term gastroscopy is required for the early detection of GTC. Patients with GTC after esophagectomy for esophageal carcinoma have a high risk of other primary carcinomas or comorbidities after ESD.

## 1. Introduction

Esophagectomy is a curative treatment for esophageal squamous cell carcinoma. The resected esophagus can be reconstructed with a gastric tube [1]; however, an increasing number of patients have been diagnosed with gastric tube carcinoma (GTC) after this procedure. Newer treatments after esophagectomy for esophageal carcinoma have also led to improved outcomes, but the incidence of GTC has also increased owing to advancements in endoscopy. Sugiura et al. reported that the prevalence of GTC has increased significantly over the years [2]. The incidence of GTC associated with *Helicobacter pylori* infection has also increased, particularly in Asian countries such as Japan [3,4].

Surgical removal of the reconstructed gastric tube in patients with GTC is associated with high mortality [2]. Endoscopic submucosal dissection (ESD), which was developed as a minimally invasive treatment for early gastrointestinal carcinoma [5], has also been used for GTC [6–8]. However, the technical difficulty of the procedure substantially increases with fibrotic changes in the suture line caused by surgical reconstruction [9], limited surgical field, and reduced maneuverability of the endoscope. There are limited reports on the role of ESD in GTC. Therefore, we examined the clinical outcomes of ESD for GTC in this study.

## 2. Materials and Methods

### 2.1. Study Design and Population

This single-center retrospective study was approved by the institutional review board of the Niigata University Medical and Dental Hospital. The study was conducted in accordance with the Declaration of Helsinki. The requirement for informed consent was waived because of the retrospective nature of the analysis.

Consecutive patients who underwent ESD for GTC after esophagectomy at our institution between 2003 and 2018 were included in this study. The indications for ESD to treat GTC were essentially based on the Japanese Classification of Gastric Carcinomas [10] and the Japanese gastric cancer treatment guidelines [11]. However, because surgical removal of the reconstructed gastric tube results in high mortality, we expanded the indication to include submucosal cancers if endoscopic ultrasonography (EUS) revealed preservation of the submucosal layer to be resected by ESD. The cancer staging for initial esophageal cancer was based on the American Joint Committee on Cancer staging manual. Atrophic gastritis of the gastric tube was diagnosed endoscopically.

### 2.2. ESD Procedure

**Figure 1:**
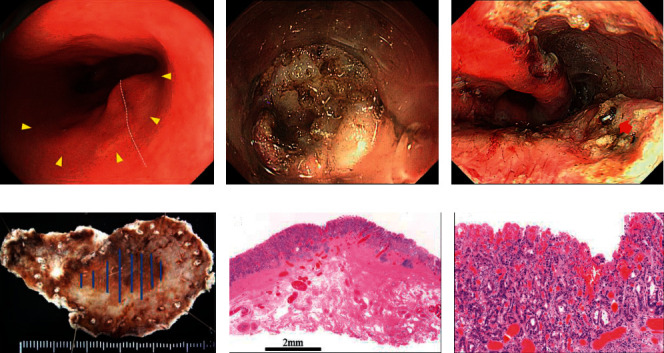
Endoscopic submucosal dissection (ESD) of gastric tube carcinoma. (a) A widespread, reddish, slightly depressed tumor (type 0-IIc) (yellow triangles) is noted straddling the suture line (white dotted line). (b) The submucosa is not clearly seen because of severe fibrosis. (c) The surgical bed is visible after ESD. A remaining surgical staple (red arrow) is seen. (d) The blue lines in the resected specimen indicate the area of the lesion. (e) The histological image demonstrates severe fibrosis in the submucosa and tumor-free vertical margins. (f) The histological type is well-to-moderately differentiated mucosal tubular adenocarcinoma.

ESD was performed by experts with more than 6 years of experience in ESD. Patients were sedated with intravenous propofol or midazolam, and blood pressure, electrocardiography, oxygen saturation, and bispectral index readings were monitored throughout the procedure. All ESD procedures were performed using an upper gastrointestinal endoscope (GIF-Q240 or GIF-Q260J; Olympus Medical Systems, Tokyo, Japan), a standard electrosurgical generator (VIO300D or ICC200; ERBE, Tübingen, Germany), and a hook knife (KD-620LR; Olympus Medical Systems). Carbon dioxide insufflation was performed during the procedure. Mucosal markings around the tumor margins were created with the hook knife. Glycerol (10% glycerin and 5% fructose) was injected into the submucosa (SM) to elevate the lesion, and bleeding vessels were coagulated using monopolar Coagrasper hemostatic forceps (ED-410LR; Olympus Medical Systems).  Figure 1 depicts a representative case of GTC on the suture line. In cases of severe fibrosis along the suture line, an ST hood short-type (DH-28GR; Fujifilm, Tokyo, Japan) and clip-with-line method was used to provide effective countertraction and good visualization [12]. The lesion was removed as a curative resection with tumor-free horizontal and vertical margins, and the histological type was well-to-moderately differentiated mucosal tubular adenocarcinoma.

### 2.3. Histopathological Assessment

The resected specimens were fastened with pins on boards and fixed in 10% formalin. Serial-step 2 mm sections were made after 24 h, stained with hematoxylin and eosin, and evaluated by pathologists. Tumor size, depth of invasion, lymphovascular invasion, presence of ulceration, and horizontal and vertical margin involvement were evaluated.

The curability of GTC with ESD was determined by the 2010 Japanese Gastric Cancer Treatment Guidelines [10]. Complete resection was defined as *en bloc* resection with tumor-free horizontal and vertical margins. Curative resection was defined as the absence of lymphovascular invasion in completely resected specimens that belonged to any of the following categories: (a) histologically differentiated-type pT1a and ulceration negative; (b) histologically differentiated-type pT1a, ulceration positive, and tumor size ≤3 cm; (c) histologically undifferentiated-type pT1a, ulceration negative, and tumor size ≤2 cm; or (d) histologically differentiated-type pT1b (SM1, <500 *μ*m from the muscularis mucosa (MM)) and tumor size ≤3 cm. Tumors with differentiated- and undifferentiated-type components were classified according to the quantitative predominance of each type. Noncurative resection was considered for specimens that did not meet the criteria for curative resection.

### 2.4. Follow-Up after ESD

Patients who underwent curative resection were monitored with annual upper endoscopic examinations, whereas repeat ESD or surgical resection was considered in patients who underwent noncurative resection. If additional treatment was difficult because of the extent of tumor invasion, upper endoscopy and computed tomography were performed every 6months.

### 2.5. Statistical Analysis

Patient characteristics, histological data, and clinical outcomes, including procedural, clinical, and long-term outcomes, were analyzed. Patient demographics, histological findings, and other continuous variables were expressed as medians and ranges, whereas noncontinuous data were expressed as percentages. Kaplan–Meier analysis was used to estimate the cumulative survival of the study population. Statistical analyses were performed using the Statistical Package for Social Science version 24 (IBM Inc., Chicago, Illinois, USA).

## 3. Results

### 3.1. Patient Characteristics and Procedural Outcomes

Patient characteristics and procedural outcomes are shown in Table 1. A total of 31 patients (29 men and 2 women) who underwent ESD for 45 GTC lesions were analyzed in this study. The median age at the time of ESD was 73 years (range, 58–84 years). Six patients had multiple lesions with synchronous occurrence, and nine patients had multiple lesions with metachronous occurrence. The median period from primary esophagectomy to ESD for GTC was 10.6 years (range, 0.8–18.2 years). The reconstruction route was retrosternal and posterior mediastinal in 16 and 15 cases, respectively. All patients in our series had atrophic gastritis. Bleeding was noted in two (6.5%) patients. No other adverse events or fatal complications, including perforation, were observed. The rates of complete and curative resection were 80.6% and 48.4%, respectively.

### 3.2. Clinical and Histopathological Findings

The histopathological findings are shown in Table 2. In terms of tumor location, there were 26, 17, and 2 lesions in the lower, middle, and upper segments of the gastric tube. Four lesions were on the suture line. Macroscopically, 34 lesions were diagnosed as type 0-IIc (slightly depressed) lesions, 10 as type 0-IIa (slightly elevated), and 1 as type 0-I (elevated) lesions. The median tumor size was 17.5 mm (range, 5–53 mm). Furthermore, 33, 2, and 10 lesions were identified as differentiated, undifferentiated, and mixed-type adenocarcinomas, respectively. The depth of tumor invasion was documented as pT1a (mucosa (M)), pT1b (SM1; <500 *μ*m below the MM in the SM), pT1b (SM2; 500 *μ*m or invasion deeper than the SM), and pT2 (muscularis propria (MP)) in 29, 3, 12, and 1 lesion, respectively. Lymphovascular invasion was observed in seven lesions. A positive horizontal margin was noted in one lesion, and positive vertical margins were noted in five lesions. The positive horizontal margin was misidentified in a poorly differentiated lesion, and the positive vertical margins were identified by invasion deeper than SM2.

### 3.3. Clinical Course after ESD and Long-Term Outcomes

**Figure 2:**
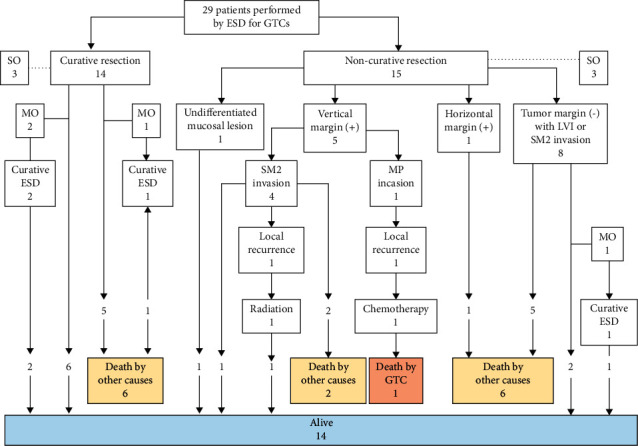
Clinical course after endoscopic submucosal dissection of gastric tube carcinoma. Curative and noncurative resections were performed in 14 and 15 patients, respectively. Among the patients who underwent curative resection, eight patients were alive, whereas six patients died of other causes. The patients who underwent noncurative resection were observed without additional surgery. Among them, two with positive vertical margins had local recurrence, one died of gastric tube carcinoma, and eight died of other causes. MO, metachronous occurrence; SO, synchronous occurrence; LVI, lymphovascular invasion; SM2, 500 *μ*m or deeper invasion from the submucosa; and MP, muscularis propria.

Twenty-nine (93.5%) patients were observed for a median period of 50 months (range, 2–168-months). Details of their clinical course are shown in Figure 2.

Curative resection and noncurative resection were performed in 14 (48.3%) and 15 (51.7%) patients, respectively. Among the 14 patients who underwent curative resection, 8 patients were alive, whereas 6 patients died of other causes (pneumonia, 3; hypopharyngeal cancer, 1; cervical esophageal cancer, 1; and sepsis due to psoas abscess, 1). Metachronous occurrence was documented in three (21.2%) patients, and curative ESD was performed in these patients. None of the patients who underwent curative resection died of GTC.

The 15 patients who underwent noncurative resection were observed without additional surgery. Among them, five patients were alive, whereas one patient died of local recurrence of gastric tube carcinoma, despite chemotherapy with 5-fluorouracil and cisplatin, and eight patients died of other causes (pneumonia, 2; heart failure, 2; intrahepatic cholangiocarcinoma, 1; hypopharyngeal cancer, 1; malignant lymphoma, 1; and pancreatic cancer, 1).

**Figure 3:**
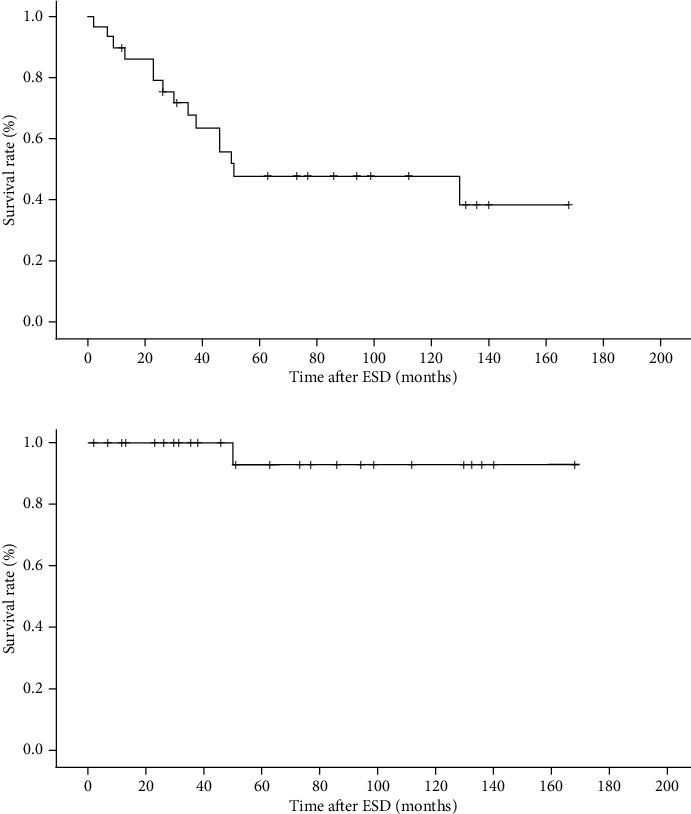
Long-term outcomes in patients who underwent endoscopic submucosal dissection (ESD) for gastric tube carcinoma. (a) Overall survival. (b) Disease-specific survival.

The 3-year and 5-year overall survival rates were 67.6% and 47.7%, respectively. The 3-year and 5-year disease-specific survival rates were 100% and 92.9%, respectively (Figure 3). Only one patient died of GTC following noncurative ESD. Of the 14 deaths due to other causes, 5 were due to other primary carcinomas (hypopharyngeal cancer, 2; cervical esophageal cancer, 1; pancreatic cancer, 1; and malignant lymphoma, 1).

## 4. Discussion

We found that ESD was safe and provided good long-term outcomes in patients with GTC. Surgical resection of the reconstructed gastric tube in GTC is invasive and associated with high mortality [2]. ESD, which is a minimally invasive treatment for early gastric cancer, plays an important role in the treatment of GTC.

The rate of complete ESD resection for GTC after esophagectomy in this study was 80.6%. Incomplete resection was noted for 1 and 5 lesions with positive horizontal and vertical margins, respectively. The lesion with a positive horizontal margin was misidentified due to the presence of undifferentiated adenocarcinoma. This lesion was >40 mm, which was not an indication for ESD. Several biopsies should have been performed to confirm the diagnosis prior to ESD. The patient subsequently died of pancreatic cancer 30months after ESD, but there was no GTC recurrence.

Of the five patients with positive vertical margins, local recurrence was noted in the patient with MP invasion who died despite chemotherapy. Local recurrence was also seen in one patient with SM invasion. Therapeutic response was noted after additional radiation in this patient, but the follow-up time was short, so remnant recurrence cannot be ruled out. No studies have demonstrated the efficacy of chemotherapy or radiation for local recurrence of GTC after ESD. The standard treatment for local massive recurrence after ESD is still total reconstruction gastrectomy; however, surgery is not widely performed because of the high risk of complications such as leakage and pneumonia [2]. Thoracoscopic and endoscopic cooperative surgery is a promising, minimally invasive option [13], and the development of other minimally invasive surgeries is expected.

Only one patient died of GTC. The disease-specific survival rate in our study was >92.9%. However, there were 14 deaths by other causes. When these deaths were considered, the 5-year overall survival rate in our study population was 47.7%. Our patients were elderly (mean age, 73 years) and diagnosed as having esophageal and gastric cancer, with an increased risk of other primary carcinomas. Moreover, they may have had other respiratory and cardiovascular diseases due to smoking, diabetes mellitus, or hyperlipidemia. We considered ESD a suitable treatment for GTC in patients with SM2 invasion when complete resection was feasible, but our data suggest that ESD should not be performed for GTC with MP invasion. When necessary, EUS should be performed prior to ESD to determine whether complete resection is possible.

While ESD was safe and effective for GTC [6,8], it was more difficult to perform in patients who underwent reconstructive surgery because the reconstructed gastric tube limits visualization of the surgical field. Moreover, severe fibrosis of the suture line and the altered anatomy of the stomach can result in food retention and fluid pooling [9]. Therefore, ESD for GTC should be performed only by fully trained endoscopists. Utilizing short-type ST hoods or applying traction with the clip-with-line method allowed good endoscopic visualization in cases of lesions on the suture line, severe fibrosis of the SM layer, or remaining staples [12,14,15]. When a lesion cannot be visualized because of fluid or blood pooling, the patient should be positioned in the opposite direction. Overall, ESD was a safe surgical option for patients with GTC because adverse events such as delayed bleeding were only noted in two cases. However, the endoscopists should exercise caution to avoid distinctive complications such as precordial skin burns, particularly when retrosternal ESD is performed [16].

All patients in this study had atrophic gastritis, which increased the risk of gastric cancer. As such, GTC should be diagnosed as early as possible [17]. Furthermore, there should be a high index of suspicion for metachronous occurrence after curative ESD. Recent remarkable advances in magnifying endoscopy and narrow-band imaging systems have allowed early diagnosis of gastric carcinomas [18]. Careful observation and management of gastric tube complications such as stomach deformity, bleeding, and fluid pooling are important. In this study, the period between primary esophagectomy and GTC diagnosis was 10.6 years; therefore, regular long-term follow-up is necessary for this patient population.

Previous studies have examined the long-term outcomes of ESD for GTC [6,8,19,20], but these studies were single-center retrospective surveys. This study was also a single-center retrospective survey; however, we examined a relatively larger number of cases over a longer follow-up period. GTC is very rare, even in highly specialized hospitals, and retrospective data from each institution is valuable because it would provide information on the efficacy and safety of ESD for GTC. Our study also analyzed the long-term outcomes in patients who underwent noncurative resection.

This study has several limitations. First, we retrospectively analyzed data collected from a single center, and the treatments were not based on any clear protocols. Second, data were collected over a long period of time, and the skills of the endoscopists may have gradually improved over this time, which may have affected the results. A multicenter prospective study would provide superior data because GTC is not frequently encountered.

## 5. Conclusions

In conclusion, our study demonstrated that ESD was safe and provided good long-term outcomes in patients with GTC. Regular long-term gastroscopy is needed for early detection of GTC. Patients with GTC after esophagectomy for esophageal carcinoma are at high risk of other primary carcinomas or comorbidities after ESD.

## Figures and Tables

**Table 1:** Patient characteristics and procedural outcomes (*n* = 31).

Patient characteristics	
Sex ratio (%)	
Male	29 (93.5)
Female	2 (6.5)
Median age, years (range)	73 (58–84)
Synchronous occurrence	6
Metachronous occurrence	9
Period from onset to esophagectomy, years (range)	10.6 (0.8–18.2)
Reconstruction route	
Retrosternal	16
Posterior mediastinum	15
Stage of initial esophageal cancer	
0	3
I	5
II	9
III	5
IV	1
Unknown	8
Atrophic gastritis	
Positive	31
Negative	0
Procedural outcomes	
Adverse events (%)	
Bleeding	2 (6.5)
Perforation	0
Complete resection (%)	25 (80.6)
Curative resection (%)	15 (48.4)

**Table 2:** Clinical and histopathological findings of GTC lesions (*n* = 45).

Location	
Upper	2
Middle	17
Lower	26
On the suture line	4
Macroscopic types	
0-I	1
0-IIa	10
0-IIc	34
Median tumor size, mm	17.5 (5–53)
Histological type	
Differentiated (tub1, tub2)	33
Undifferentiated (por, sig)	2
Mixed	10
Invasion depth	
pT1a (M)	29
pT1b (SM1)	3
pT1b (SM2)	12
pT2 (MP)	1
Lymphovascular invasion	
Positive	7
Negative	38
Horizontal margin	
Positive	1
Negative	44
Vertical margin	
Positive	5
Negative	40

tub1, well-differentiated adenocarcinoma; tub2, moderately differentiated adenocarcinoma; por, poorly differentiated adenocarcinoma; sig, signet-ring cell carcinoma; M, mucosal; SM1, <500 *μ*m below the muscularis mucosa into the submucosa; SM2, 500 *μ*m or deeper invasion from the muscularis mucosa into the submucosa.

## Data Availability

The datasets collected and analyzed in this study are available from the corresponding author on reasonable request.
